# Vitrification and Subsequent *In Vitro* Maturation of Mouse Preantral Follicles in Presence of Growth Factors

**Published:** 2014-10-04

**Authors:** Zahra Oryan Abkenar, Roya Ganji, Amir Eghbal Khajehrahimi, Mohammad Hadi Bahadori

**Affiliations:** 1 Department of Biology, Damghan Branch, Islamic Azad University, Damghan, Iran; 2 Faculty of Biology, Tarbiat Moallem University, Tehran, Iran; 3 Branch of North of Tehran, Islamic Azad University, Tehran, Iran; 4 Cellular and Molecular Research Center, Faculty of Medicine, Guilan University of Medical Science, Rasht, Iran

**Keywords:** Vitrification, Mouse Preantral Follicle, *In Vitro* Maturation, Epidermal Growth Factor, Fibroblast Growth Factor

## Abstract

**Objective:**

Cryopreservation of ovarian tissue or follicles has been proposed as an alternative method for fertility preservation. Although successful vitrification of follicles has been reported in several mammalian species, the survival rate is generally low. The aim of this study was to investigate the effects of fibroblast growth factor (FGF) and epidermal growth factor (EGF) on *in vitro* preantral follicle development after vitrification.

**Materials and Methods:**

In this experimental study, preantral follicles with diameter of 150-180 µm were mechanically isolated from ovaries of 18-21 days old NMRI mice. Follicles were vitrified and warmed, then cultured in a-minimal essential medium (α-MEM) without growth factor supplementation as control group (group I), while supplemented with 20 ng/ml FGF (group II), 20 ng/ml EGF (group III), and 20 ng/ml FGF +20 ng/ml EGF (group IV). After 12 days, human chorionic gonadotrophin (hCG)/EGF was added to culture medium, and after 18-20 hours, the presence of cumulus oocyte complexes (COCs) and oocyte maturation were assessed. The chi-square (Χ^2^) test was used to analyze survival and ovulation rates of the follicles.

**Results:**

Our results showed that the rate of metaphase II (MII) oocytes in FGF group increased in comparison with control and other treatment groups (p<0.027), but there was no difference between control with EGF and EGF+FGF groups in oocyte maturation rate (p>0.05). There was a significant decrease in survival rate of follicles in EGF+FGE group in comparison with other groups (p<0.008). After *in vitro* ovulation induction, the follicles in EGF group showed a higher ovulation rate (p<0.008) than those cultured in other groups.

**Conclusion:**

FGF has beneficial effect on oocyte maturation, and EGF increases COCs number *in vitro*. Combination of EGF and FGE decreases the number of survived follicles.

## Introduction

Cryopreservation of ovarian tissue or follicles has been proposed as an alternative method for fertility preservation (1-3). After isolation of follicles from ovarian tissue using enzymatic or mechanical techniques, follicles would require further *in vitro* maturation (4-6). The penetration of the cryoprotectant agent into the follicular structure is easier compared to ovarian tissue (4). In addition, the assessment of follicles after thawing is easier than whole ovarian tissue (7). For these reasons, cryopreservation of isolated ovarian follicles seems to be more attractive option than other methods (8). Although vitrification is considered as a simple cryopreservation method, the requirement steps for high concentrations of cryoprotectant cause osmotic and toxic damage to cells (9).

It is demonstrated that culture of follicles isolated from frozen/thawed ovarian tissue produces mature oocytes, but the diameter of these follicles is smaller than that of fresh ones (10). The primordial follicle is the earliest form of follicle in the ovaries that initiates the next phase of development under different unknown signals (11). Only a few follicles reach to the ovulation stage. The mechanism by which the primordial follicles develop to preantral stage is yet unclear. 

The immature oocytes are not injured during the process of vitrification due to following factors: small size, few developments, few organelles, absence of zona pellucid and low metabolism. Culture of ovarian follicles is an alternative method for fertility treatment. Recently various culture systems for preantral follicles and oocyte-granulosa cell complexes have also been proposed (12-13).

The factors and mechanisms involved in this process are not yet fully understood. Irrespective of gonadotropin involvement, there is good evidence suggesting that local regulatory factors are implicated in this temporal and spatial process (14, 15).

The fibroblast growth factors (FGF-4s), as heparinbinding single chain polypeptides, have a crucial role in development, cell growth and tissue repair

They stimulate the ovarian granulose cell differentiation (16), the expression of the luteinizing hormone (LH) receptors by granulose cells, and the proliferation of ovarian germinal cells (17). Epidermal growth factor (EGF) results in cellular proliferation and survival (18). Furthermore, EGF plays a role in oocyte *in vitro* maturation (19), while stimulates the proliferation of granulosa cells *in vivo* and *in vitro* (20). In human oocytes, the expressions of EGF and its receptor is detected in follicles at preantral stage (21).

The aim of this study was to investigate the effects of fibroblast growth factor (FGF) and epidermal growth factor (EGF) on *in vitro* preantral follicle development after vitrification.

## Materials and Methods

### Animals

In this experimental study, all female mice used in this study were obtained from the Razi Institute, Karaj, Iran. The animals were housed individually in an air-conditioned controlled room at 23-25˚C and under a 12 hour light: 12 hours dark cycle (6 am: 6 pm), fed a commercial diet, and given water ad libitum. All the animal experimentations were approved by the Animal Ethics Committee at the Guilan University of Medical Sciences (GUMS).

### Isolation of preantral follicles

Ovaries of prepubertal Naval Medical Research Institute mice (aged 18-21 days) were aseptically removed from the animals after being killed and placed in rewarmed isolation medium, consisting of alpha-minimum essential medium (á-MEM; Gibco-Invitrogen, Germany) supplemented with 10% v/v fetal bovine serum (FBS, Sigma, Germany) and 100 IU/ml penicillin +100 ìg/ml streptomycin (Sigma, Germany). The ovaries were mechanically dissected using fine hypodermic needles (a 26-gauge). Follicles with a diameter in the range 150-180 ìm were then collected.

### Vitrification procedure

Preantral follicles were vitrified using an ethylene glycol (EG) and dimethyl sulfoxide (DMSO, Sigma, Germany) based on the protocol. The base media for the preparation of equilibration and vitrification solutions was α-MEM + 20% FBS. Follicles were equilibrated for 3 minutes in 7.5% equilibration solution containing 7.5% EG + 7.5% DMSO followed by a 30-40 second incubation in vitrification solution containing 15% EG + 15% DMSO + 0.5M sucrose. As soon as cellular shrinkage was observed, five preantral follicles were aspirated and placed on the tip of the cryolock (Biodiseno, USA). Cooling of the preantral follicles was done by direct contact with liquid nitrogen. The cryolocks were stored in liquid nitrogen for 30 days. All vitrification procedures were performed at room temperature.

### Warming

For warming, the cryolocks containing vitrified preantral follicles were held in air for 20 seconds at room temperature. Then they were exposed with warming solutions by serial dilution in base medium with decreasing concentration of sucrose from 1 M to 0.5 M and 0.5 M to 0 M for 1-3 and 40-45 minutes, respectively. All procedures were carried out at room temperature, but the last step in base medium was performed at 37˚C.

### Evaluation of immediate post-warming survival rate

Survival of vitrified/warmed follicles was assessed microscopically based on morphology of the follicle under a stereomicroscope followed by inverted microscope (IX71, Olympus, Japan). A follicle was considered to be intact if it possessed an oocyte surrounded by a complete tight collar of granulosa cells (GCs). Follicles with partially or completely naked oocytes or large spaces within the granulosa-oocyte complex were graded as damaged. Any dark artisticlooking follicles were also graded as damaged. Only intact preantral follicles were selected for further in vitro culture (IVC).

### In vitro culture of vitrified-warmed preantral follicles

Vitrified/warmed preantral follicles were individually cultured in 20 μl droplets of maturation medium containing α-MEM supplemented with 1% insulin, transferrin, and selenium ITS (Invitrogen, USA); 100 mIU/mL recombinant human follicle stimulating hormone (rhFSH, (Gonal-f, Merck Serono, Switzerland), and 5% FBS (Sigma, Germany). The follicles were cultured for 12 days at 37˚C in an atmosphere of 5% CO_2_ in 60×15 mm Petri dish (Falcon, USA) covered with 5ml mineral oil (Sigma, Germany).

### Experimental groups

Vitrified/warmed preantral follicles were cultured in maturation medium without growth factor supplementation as control group (group I), while supplemented with 20 ng/ml FGF (group II), 20 ng/ml EGF (group III) and with combination of 20 ng/ml EGF and 20 ng/ml FGF (group IV).

### Ovulation induction

On day 12 of culture, 1.5 IU/ml recombinant human chorionic gonadotrophin (rhCG) and 5 ng/ml recombinant epidermal growth factor (rEGF) were added to the cultures as *in vitro* ovulatory stimulus. Optimal nuclear maturation rate was achieved after 18-20 hours of induction (22).

### Statistical analysis

We used the chi-square (χ2) test to analyze survival and ovulation rates of the follicles and the nuclear maturation of the oocytes. Data analysis was performed using Statistical Package for the Social Sciences (SPSS, SPSS Inc., Chicago, IL, USA) version 16.

## Results

### Survival of vitrified-warmed preantral follicles

**Fig 1:**
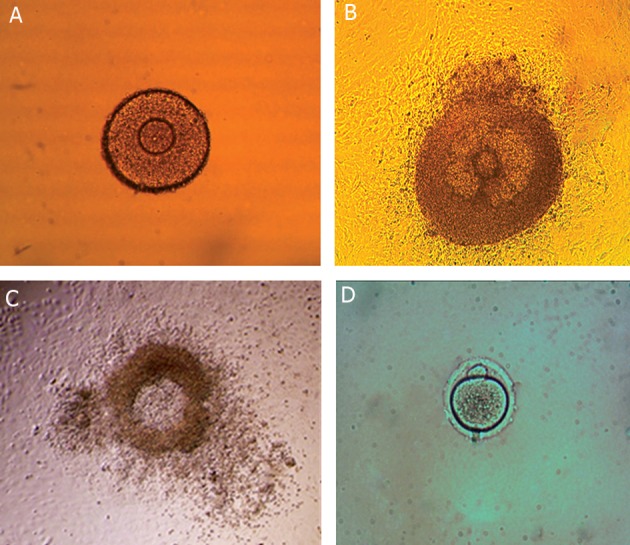
Preantral follicle on day 1 of culture (EGF group) (×100) (A), an antral follicle after 10 days of culture (EGF group) (×100) (B), an antral follicle stimulated with hCG/EGF showing COC extraction (EGFgroup) (×40) (C) and MII oocyte after in vitro ovulation (EGF group) (×400) (D).

On day 1 of culture, the healthy follicles had attached to the culture dish (Fig 1A).

On day 12, there was a significant (p<0.008) decrease in the number of survived follicles (67.8%) in group III (FGF+EGF) in comparison with control group. There was no significant difference (p>0.05) between control, FGF and EGF groups, while their survival rates were 84.6, 79.3 and 77.3%, respectively.

### In vitro ovulation of vitrified-warmed preantral follicles

At the end of the culture (Fig 1B), the follicles were stimulated by rhCG and rEGF to induceovulation. Cumulus-oocyte complexs (COCs) were expanded and released after 18-20 hours of stimulating follicles (Fig 1C).

**Fig 2:**
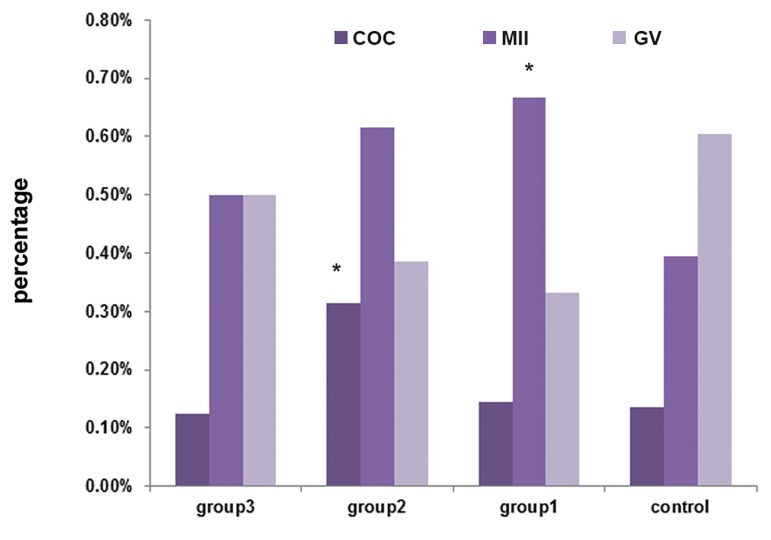
Oocyte maturation and *in vitro* ovulation. High maturation rate was observed in FGF group and high ovulation rate was observed in EGF group. Statistical differences are indicated above the columns and *; P<0.05.

Our results revealed significant differences (p<0.05) in the proportion of ovulated COCs between the control and EGF groups. The highest percentage of released COCs (31.4%) was observed in group III with 20 ng/ml EGF as compared to other groups (13.6, 14.5, and 12.5% in control, II and IV groups, respectively) (p˂0.008) (Fig 2).

### Maturation state of oocytes

The percentage rates of oocytes reaching to MII stage (Fig 1D) were 39.5, 66.7, 61.5, and 50% in the control, II, III and IV groups, respectively. We observed significant maturation rate in 20 ng/ml of FGF (66.7%, p<0.027) compared with those cultured in the other groups (Fig 2).

## Discussion

Freezing and thawing procedures can decrease the growth rate of oocytes and granulose cells in the follicles.

Cryopreservation depends on survival rates of the granulosa cells and oocytes and on the maintenance of gap junctions between the granulosa cells and oocytes (23). Recent studies have indicated high rate of follicular survival with normal morphology after cryopreservation (23).

The type of the cryo-device may affect the process of vitrification. The carriers, such as cryoloop (24), cryotop (25), open pulled straw (26), and EM grid (27), load a very minimal amount of cryopreservation solutions and increase the rate of cooling that is important in the vitrification process.

Among these devices, cryotop has been successfully applied for ovarian tissue vitrification (25, 28). The majority of cryo-devices in vitrification of isolated ovarian follicles include the open carrier systems (29, 30).

We preferred to use cryolock as a derivative of cryotop. Our results demonstrated that about 84% of follicles were morphologically intact immediately post warming, while 13.6% survived to the end of the IVC interval period in the control group. For normal development of ovarian follicles *in vivo*, a combination of endocrine, autocrine, and paracrine signals growth factor signals is required to work closely .

FGFs are involved in various biological processes during folliculogenesis as follows i. primordial follicle activation, ii. regulation of granulosa and cumulus cell mitosis, apoptosis and glycolysis (31-33), iii. the expression of LH receptors induced by granulose cells, and iv. proliferation of ovarian germ cells Zhang and Ealy demonstrated that COCs incubated with FGF2 showed an increase in the percentage of oocytes with an extruded polar body as compared to controls, while no significant differences in polar body extrusion rates were detected between FGF2 treatments (34).

Oocyte maturation of IVC method is determined by nuclear maturation and detected by the first polar body (35).

The results of the present study show clearly that FGF-treated follicles produce more MII oocytes; however, we did not observe any positive effect of FGF on survival and ovulation rates of cultured follicles.

FGF is a molecule with multiple functions in the body. In most *in vitro* culture, FGF promotes proliferation of granulosa cells of various species (15, 36-39). Despite of its presence in oocyte and granulosa cells of most follicle stages, the expression level of FGF in different stages is not clear (40-45). In a study by sharma et al. (46), survival, growth, antrum formation and steroidogenesis are stimulated by insulin growth factor-I and bFGF, whereas tumor growth factor-alpha + tumor growth factor -beta1 inhibited growth and survival of PFs which led to induced oocyte apoptosis in buffalo preantral follicles.

In combination with gonadotropins, FGF-4 increased cumulus expansion and number of metaphase II-stage oocytes in *in vitro* culture (22). It has been reported that EGF increases the proportion of metaphase II stage oocytes of COCs isolated from small follicles (47). In COCs of mouse and pig, EGF-like growth factors is regulated by autocrine mechanism (48, 49). A significant positive EGF on IVM of oocytes was reported in pigs (43, 45). Our finding showed that EGF enhances cumulus expansion and post-thaw COC formation.

Furthermore, expansion of cumulus cells is induced by any changes occurring in level of gonadotropins, growth factors, steroids, and other factors secreted by the oocyte (50). The presence of EGF and a related ligand, transforming growth factor-á (TGF-á), in the human and mouse follicular fluid indicates the participation of EGF in regulation of oocyte maturation (51).

The results of our study showed that the use of 40 ng/ml EGF and FGF simultaneously reduced the number of surviving follicles.

EGF stimulates oocyte maturation through destroying communications between oocyte and cumulus cells (52) or through the signaling pathways promoting oocyte maturation (53).

## Conclusion

The inclusion of EGF and FGF at a concentration of 20 ng/ml in mouse leading to vitrified follicle culture system has no effect on follicle survival. Furthermore, 20 ng/ml FGF signi.cantly increases oocyte maturation capacity, where 20 ng/ml EGF only in.uences ovulation *in vitro*. Combination of FGF and EGF has no effect on survival rate, oocyte maturation and ovulation.
